# Physician and Patient Preferences for Oral Anticoagulation Therapy Decision Making in Atrial Fibrillation: Results From a National Best–Worst Scaling Survey in Türkiye

**DOI:** 10.1002/clc.70038

**Published:** 2024-12-09

**Authors:** K. Kılıckesmez, D. Aras, M. Degertekin, N. Ozer, B. Hacibedel, K. Helvacioglu, U. Koc, B. Ozdengulsun, E. Dundar Ahi, O. Ergene

**Affiliations:** ^1^ Department of Cardiology Cemil Taşcıoğlu Research and Training Hospital İstanbul Türkiye; ^2^ Department of Cardiology Medipol University İstanbul Türkiye; ^3^ Department of Cardiology Yeditepe University İstanbul Türkiye; ^4^ Department of Cardiology Hacettepe University Ankara Türkiye; ^5^ Pfizer İstanbul Türkiye; ^6^ Department of Cardiology Dokuz Eylül University İzmir Türkiye

**Keywords:** atrial fibrilation, NOACs, oral anticoagulation, patients' preferences, treatment decision

## Abstract

Atrial fibrillation (AF) is the most common cardiac dysrhythmia and a common cause of ischemic stroke. Stroke prevention with oral anticoagulation (OAC) is the cornerstone of AF management. Patients and their treating physicians may have different views on different attributes of OACs. The objective of this study was to quantify the relative importance that patients and physicians in Turkey place on different OAC attributes when making treatment decisions in AF. A cross‐sectional survey was administered to AF patients (≥ 50 years) receiving OAC and practising cardiologists, including residents with ≥ 2 years of experience in Turkey. For both patients (*N* = 230; 50% male) and physicians (*N* = 194; 74% male), the most important attributes for OAC treatment decision making in AF were “success in preventing stroke” (57% and 73.9% or overall importance, respectively) and “risk of major bleeding” (20% and 23.4%, respectively). For patients, other attributes were much less important, but not altogether unimportant: reversal agent availability (7%), monitoring (5%), food or drug interactions (3%), minor bleeding (3%), and ease of swallowing (2%). For physicians, among the other attributes, only the need for monitoring (1.3%) had a relative importance of > 1%. For all Turkish participants, efficacy and safety were found to be the most important attributes influencing OAC choice in AF with these two attributes accounting for 77% and 97.3% of overall importance for patients and physicians, respectively. Certain considerations, especially reversal agent availability and monitoring appear to be more important to patients than to physicians This is the first study to use BWS to quantify patient and physician preferences for OAC treatments in AF in Turkey.

## 1 Introduction

With a prevalence of 1%–3% in the general population, atrial fibrillation (AF) is the most common persistent cardiac dysrhythmia and one of the most common causes of ischemic stroke [1, 2]. In particular, the risk of ischemic strokes is up to fivefold higher in AF patients than in the general population which results in significant clinical and economic burden [3]. The Copenhagen Stroke Study revealed that AF was associated with a 70% increase in mortality, a 40% decrease in discharge rate, a 20% increase in the length of hospital stay, and a marked increase in impairment and disability among survivors [4]. Moreover, subclinical atrial tachyarrhythmias were independently associated with a substantial increase in the risk of ischemic stroke or systemic embolism [5].

Stroke prevention with oral anticoagulation (OAC) therapy is the cornerstone of the management of patients with atrial fibrillation. “Avoid stroke/anticoagulation” is the “A” of the Atrial Fibrillation Better Care (ABC) pathway which is recommended and encouraged in the European Society of Cardiology (ESC) guidelines [6]. OAC therapies including vitamin K antagonists (VKA), such as warfarin, and non‐VKA oral anticoagulants (NOACs), significantly reduce the risk of stroke (by at least 64%) and death (by at least 26%) compared with control or placebo [6, 7, 8]. Structured, clinical, risk‐score−based assessment of individual thrombo‐embolic risk, using the CHA2DS2‐VASc Score, should be performed as the first step in optimal thrombo‐embolic risk management in AF patients. Patients with AF and risk factors for stroke need to be treated with OAC and, if patients are eligible, NOACs should be preferred over VKAs [6].

Clinical guidelines recommend taking into account patients' views and preferences to enable shared decision‐making and develop strategies for improved awareness and better management of AF [3]. To support these objectives, understanding the preferences of AF patients for anticoagulation therapy and physicians' attitudes toward prescribing OAC to patients with AF is critical. A systematic literature review revealed that the preferences of AF patients for preventive anticoagulant treatment may vary from the principles of clinical guidelines or the view of physicians [9]. While effectiveness and safety have been found as the most important patient attributes of OAC therapy in many studies, some data has also shown that AF patients prefer the anticoagulant agents with once‐daily frequency, no requirement for bridging, and no interactions with food [10, 11]. Reports from Canada, the United Kingdom, and the United States revealed that physician self‐reported comfort level, observed clinical benefits and risks, patient comfort and satisfaction, and product costs are common factors affecting physicians' choice of anticoagulants in AF management [12, 13, 14].

Previous studies from Türkiye reported that oral anticoagulant therapy was used by 40% of 1745 non‐valvular AF patients, about 30% of patients were undertreated while 7%–10% of AF patients were overtreated with NOACs [15, 16, 17]. The ASPECT‐NOAC study aimed to assess patient characteristics, disease knowledge, and treatment adherence in AF patients who recently started NOAC therapy. The study found that the average level of knowledge about AF was less than 50%, and there was a significant variability in knowledge levels among patients [18]. The multicentric NOAC‐TR study illustrated that more than 50% of patients have low compliance with OAC treatment in Türkiye [19]. Inadequate knowledge of patients on NVAF, the inability of physicians to spend adequate time with patients, and reimbursement conditions were reported to affect adherence and persistence to NOAC treatments in Türkiye [20].

To date, no study has aimed to quantify patients' preferences for specific OAC attributes in AF management, nor to determine the factors that influence physicians' OAC prescribing decisions in Türkiye. The objective of this study was to quantify the relative importance of different OAC attributes to patients and physicians in Türkiye when making treatment decisions.

## 2 Materials and Methods

### 2.1 Study Design and Population

This was a national, descriptive, cross‐sectional survey study of 230 patients with non‐valvular AF, aged ≥ 50 years and receiving OAC for stroke prevention, and 194 currently practicing cardiologists, including cardiology residents with a minimum 2 years of experience. The patients enrolled in this study were patients of five centers in Türkiye; Prof. Dr. Cemil Taşcıoğlu City Hospital and Yeditepe University Hospital in İstanbul, City Hospital and Hacettepe University Faculty of Medicine Hospital in Ankara and Dokuz Eylül University Faculty of Medicine in İzmir. The patients consisted of outpatient clinic patients. There were no exclusion criteria for either group. By including patients currently being treated with OACs, the study aims to capture preferences based on treatment experience. While the majority of the best–worst scaling (BWS) studies provided no justification for their choice of sample size, sample sizes/quotas in our study were determined according to feasibility estimates within the patient and physician groups [21].

Physician participants were invited by investigators and recruited by a contracted CRO and patient participants were recruited by the same CRO at investigators' clinics. Written informed consent was obtained from all participants and ethics committee approval was obtained from Dokuz Eylül University, İzmir, Türkiye. The survey was conducted in accordance with the principles of the Declaration of Helsinki.

### 2.2 Interview Questionnaires and Survey Tools

A two‐part questionnaire was used for the patient survey which included both demographic and clinical questions and a BWS exercise. Demographic and clinical characteristics included age, gender, education, socioeconomic status, number of people in the household, and time since AF diagnosis. BWS exercise included the following factors associated with anticoagulation treatment: success in preventing stroke, risk of major bleeding (bleedings that would require admission to emergency care), risk of minor bleeding (bleedings that would not require admission to emergency care, such as gingival bleeding, bruising), daily usage frequency, availability of treatments that can reverse anticoagulation in case it is necessary (such as bleeding, overdose, etc.), need for monitoring, restrictions due to food and drug interactions, duration of the product in the market, ease of swallowing and requirement to be taken with food or not [22, 23, 24, 25, 26] (Appendix 1). Patients were stratified according to socioeconomic status. Object‐case BWS was used to assess the relative importance of the 10 OAC characteristics. BWS is a survey method for assessing individuals' priorities. It identifies the extremes – best and worst items, most and least important factors, biggest and smallest influences – among sets [27]. In object‐case BWS, a respondent is presented with a series of questions. In each question, a subset of the full set of characteristics was asked to indicate which is most preferred and which is least preferred [28].

For participating physicians, a three‐part questionnaire included demographic and practice characteristics (academic affiliation, years of experience, gender, type of institution, city of institution) and a BWS exercise using the same factors used in the patient questionnaire. In the third final part, physicians were also asked if their rankings would differ based on patient subgroups and if the response was yes they were then asked to reconsider their rankings for each patient subgroup (patients with a history of stroke/transient ischemic attack (TIA), elderly patients over 80 years of age, patients with acute coronary syndrome (ACS) and percutaneous coronary intervention (PCI) in addition to AF, male patients with the CHA_2_DS_2_‐VASc Score ≥1, female patients with the CHA_2_DS_2_‐VASc Score ≥ 2, patients with renal function impairment (glomerular filtration rate [GFR] < 60) and patients with minor bleeding).

Both patient and physician surveys were administered from October 2021 to February 2022. Patient surveys were conducted in person or by Computer‐Assisted Telephone Interviewing (CATI) technique, whereas clinician surveys were performed by the CAWI technique. In our survey, each respondent was presented with a series of questions each including 10 items. The sets of items in each question and the series of questions were determined by a balanced incomplete block experimental design. By using this design, we could choose the subset size to be less than or equal to the number of samples that a participant can comfortably evaluate in one session; a natural consequence being that now a larger range of items may be investigated in the same experiment [29].

### 2.3 Statistical Analysis

To gain a better insight for data of the questionnaire, and descriptive analyses were conducted. For demographics and nominal variables, frequencies and percentages, and for scale and interval variables descriptive (mean, standard deviations, etc.) were reported. An Independent sample *t*‐test was used to understand the statistical differences between the two groups. Kendall rank correlation was used to test the similarities in the ordering of data when it is ranked by quantities. As additional analysis, other parametric and non‐parametric analyses were conducted. The quantum tabulation tool SPSS software was in addition to Statistical Package for the Social Sciences (SPSS).

Object‐case BWS was used to assess the relative importance of 10 OAC characteristics including stroke prevention, bleeding risks, need for monitoring, availability of reversal agent, and administration‐related characteristics. BWS is a discrete choice task in which a person is asked to indicate the “least preferred” item in a choice set, in addition to indicating the (traditional) “most preferred” item [27, 28]. Maximum difference scaling (MaxDiff) – also known as the BWS – an approach that involves choice modeling (or discrete choice experiment – “DCE”) was completed using Sawtooth Software Lighthouse Studio to measure the relative importance of OAC characteristics according to patients and physicians [27, 28, 29, 30]. Relative importance was estimated as the proportion of overall importance.

This method of research allows respondents to express their opinion toward a preference in a natural and intuitive way (“Which of these is the most important for you?”) but is also able to deduce numeric, comparative values for each option on screen. Since the same question is asked for a statistically modeled set of changing screens, inferences such as A > B > D > C can be safely calculated. Hierarchical Bayes Estimation was used in calculating said “utility values,” which is the most advanced method of estimating preference utilities to date.

## 3 Results

### 3.1 Patient Data

#### 3.1.1 Demographics

A total of 230 patients (114 females and 116 males) answered the survey. Of these, 70% were married, 28% were divorced/widowed, and 2% were single. The educational background of the patients was classified as 18% university and above, 12% high school, 50% primary and secondary school and under, and 20% no formal education. The unemployment rate was 89% among responders. Ninety percent of the data was gathered from the three largest and diverse cities of Türkiye (İstanbul, Ankara, and İzmir). Out of 230 responders, 6% were in the highest socioeconomic group whilst 12% were in the lowest band. Arterial hypertension was the most common comorbidity, followed by diabetes mellitus and cardiac failure. While hypertension and rheumatological disorders were more frequent in females, other comorbidities were more prevalent in males. The stroke rate was significantly higher in the top two highest socioeconomic groups compared to the middle and lowest two ones. Although hypertension, diabetes, and cardiac failure were more common in 60‐ to 69‐year‐old patients, rates of previous stroke history and liver disease were higher in 50‐ to 59‐year‐old patients and gradually decreased by age. Overall, 98% of patients were on treatment with oral anticoagulants (apixaban, rivaroxaban, dabigatran, edoxaban, warfarin). The proportion of patients receiving OACs for up to 2 years was 33% while the patient percentage using OACs more than 20 years was only 8%. Patients in lower socioeconomic groups were using OACs significantly higher than the lower ones (Table 1).

**Table 1:** Demographic and baseline characteristics of survey patients with non‐valvular AF, aged ≥ 50 years and receiving OAC for stroke prevention in Türkiye (*n* = 230).

Participant characteristics		Numbers	Percentage
**Gender**	Female	114	50
	Male	116	50
**Age**	50–59 years	31	14
	60–69 years	63	27
	≥ 70 years	136	59
**Marital status**	Single	5	2
	Married	160	70
	Divorced/widowed	65	28
**Working status**	Employed	25	11
	Unemployed	205	89
**Education status**	University and above	42	18
	High school	29	12
	Primary and secondary school	114	50
	No formal education	45	20
**Residency city**	İstanbul	116	51
	Ankara	52	23
	İzmir	36	16
	Other	24	10
**Socioeconomic status**	A	14	6
	B	19	8
	C1	38	17
	C2	59	26
	D	72	31
	E	28	12
**Comorbidities**	Arterial hypertension	128	56
	Diabetes Mellitus	56	24
	Cardiac failure	54	23
	Rheumatologic diseases	37	16
	Chronic lung disease	24	10
	Chronic kidney disease	13	6
	Chronic liver disease	7	3
	Previous stroke	11	5
**OAC use**	Up to 2 years	76	33
	3–5 years	57	25
	6–10 years	26	11
	11–20 years	34	15
	More than 20 years	18	8
	Did not recall	19	8

*Note:* Socioeconomical (SEC) status is defined by Turkish Researchers' Association.

#### 3.1.2 Factors Affecting Patients' Preference on Anticoagulation Treatment

**Figure 1:**
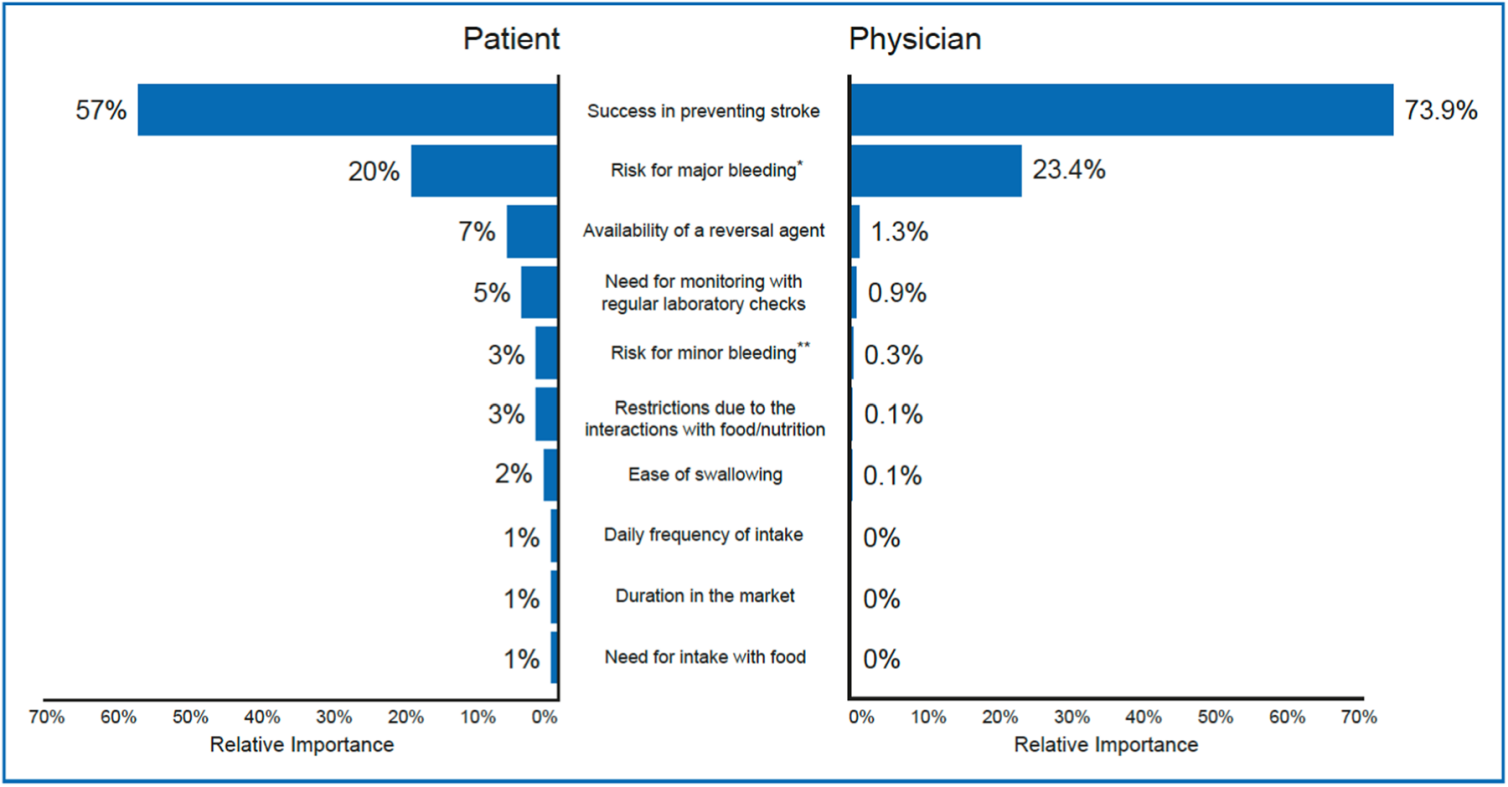
Relative importance estimates of patients (*n* = 230) and physicians (*n* = 194) on anticoagulation treatment attributes in Türkiye.

Relative importance estimates for patients are shown in Figure 1. Success in preventing stroke was chosen as the most important factor (57%), whereas lower risk for major bleeding was seen as the second priority (20%). Availability of a reversal agent (7%), need for monitoring with regular laboratory checks (5%), likelihood for minor bleeding (3%), restrictions due to the interactions with food/nutrition (3%), ease of swallowing (2%), daily frequency of intake (1%), need for intake with food (1%), duration in the market (1%) were less important concerns for patient preference (*n* = 230).

**Figure 2:**
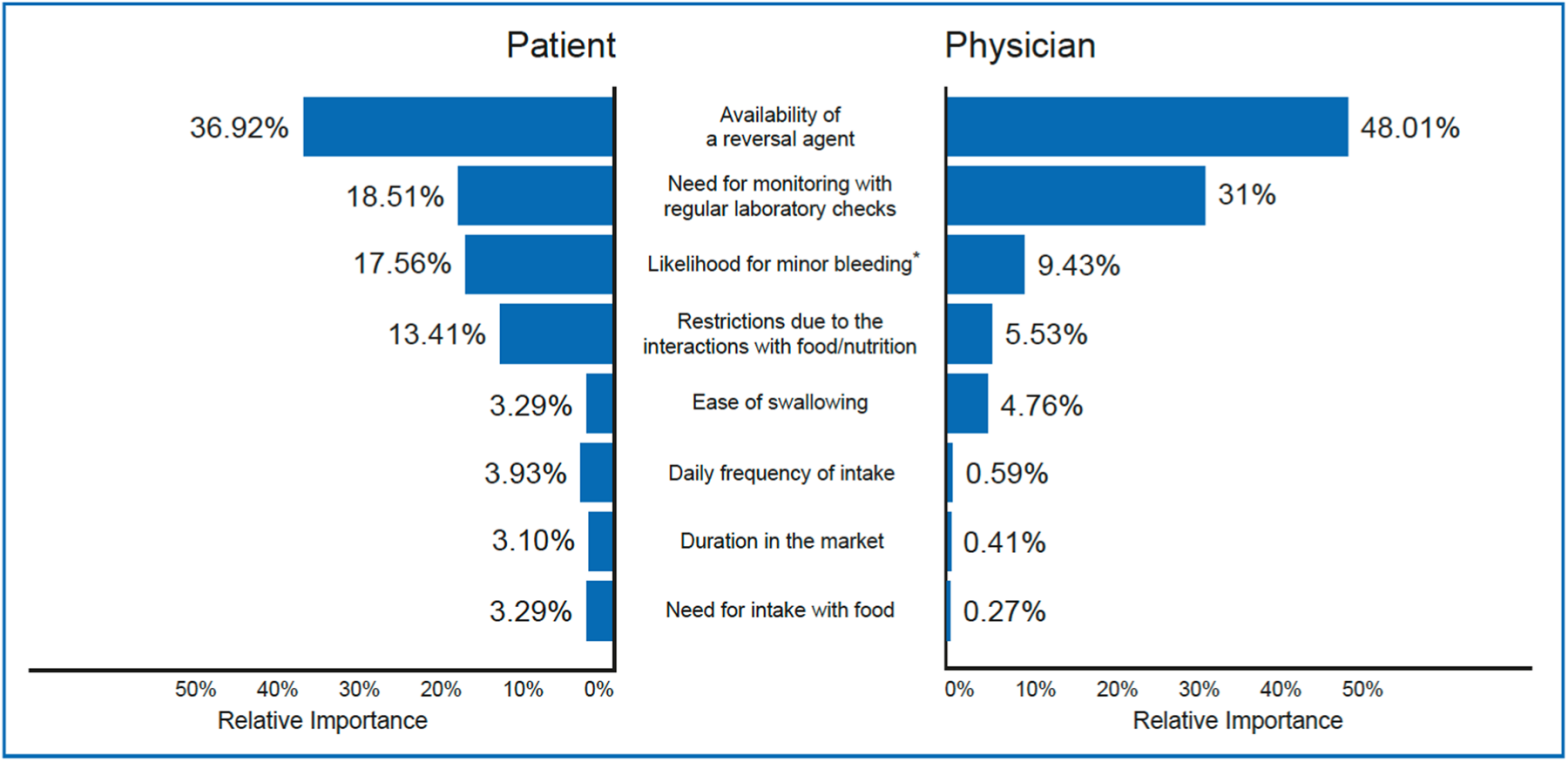
Relative importance estimates of patients (*n* = 230) and physicians (*n* = 194) on anticoagulation treatment attributes in Türkiye, assuming the relative importance of “success in stroke prevention” and “major bleeding risk” are equal.

Because two attributes dominate decision making for OAC treatment, we conducted a post‐hoc analysis to specifically understand the relative importance of other attributes, assuming “success in stroke prevention” and “major bleeding risk” were equal among alternative OAC options. In this secondary analysis, “availability of a reversal agent” was found to be the most important factor among the remaining attributes, with a relative importance of 36.92% (Figure 2).

### 3.2 Physician Data

#### 3.2.1 Demographics

86% of the physicians were from hospitals where training facilities are present; 105 of them were from research and teaching hospitals, 62 were from university clinics, 19 were from private hospitals/clinics, and 8 of them were from state hospitals without training facilities (*n* = 194). While 74% of physicians were males, only 26% of them were females, 180 out of 194 were in the three largest cities of Türkiye (110 in İstanbul, 54 in Ankara, and 16 in İzmir); 34% of the physicians were cardiology residents with a minimum 2 years of experience, 41% were cardiology consultants and 25% were academics including professors, associate professors, and assistant professors with ratios 5%, 16%, and 4%, respectively (Table 2).

**Table 2:** Demographic, professional, and geographical characteristics of survey physicians who are practicing cardiologists (including cardiology residents with a minimum of 2 years of experience) in Türkiye (*n* = 194).

Participant characteristics		Numbers		Percentage	
Gender	Female	50		26	
	Male	144		74	
Title	Professor	10		5	
	Associate professor	30		16	
	Assistant professor	8		4	
	Consultants	80		41	
	Clinical residents with minimum 2‐year experience	66		34	
Working place	Research and Teaching Hospitals	105		54	
	University clinics	62		32	
	Private hospitals/clinics	19		10	
	State hospitals	8		4	
Residency city	İstanbul	110		57	
	Ankara	54		28	
	İzmir	16		8	
	Other	14		7	

#### 3.2.2 Factors Affecting Physicians' Preference on Anticoagulation Treatment

Relative importance to physicians of anticoagulation treatment attributes is shown in Figure 1. The success of preventing stroke was reported as the most important factor affecting their treatment preference where the risk for major bleeding was the second most remarkable consideration point consistent with the patient preference ratings. The relative importance of the need for monitoring with regular laboratory tests received a 1.3% share of preference rating, whereas availability of a reversal agent, the likelihood for minor bleeding, restrictions due to the interactions with food/nutrition, ease of swallowing, daily frequency of intake, need for intake with food and duration in the market received a lower share of preference with less than 1% relative importance rate for each.

Using the same approach as in the analysis of the patient data, we conducted a follow‐up analysis of the physician data to specifically understand the relative importance of other attributes, assuming that the two dominant items, “success in stroke prevention” and “major bleeding risk”, were equal among alternative OAC options. In this secondary analysis, “availability of a reversal agent” and “need for monitoring” were the most important attributes, with the estimated relative importance of 48.01% and 31%, respectively (Figure 2).

#### 3.2.3 Physicians' Preferences According to Patient Subgroups

While 80% of the physicians would not have changed their preferences based on differences in patient characteristics, 20% (*n* = 194) of physicians reported that they would have provided different responses if they were considering treatment for patients over 80 years of age or patients with renal impairment. With regard to other patient characteristics, 18% of physicians would respond differently if that patient has Acute Coronary Syndrome (ACS) or Percutaneous Coronary Intervention (PCI) in addition to AF, 12% if the patient were male with CHA_2_DS_2_‐VASc Score ≥1 or female patients with CHA_2_DS_2_‐VASc Score ≥ 2, 7% if the patients had a history of stroke/TIA, and 7% if the patient had minor bleeding (*n* = 194).

## 4 Discussion

Atrial fibrillation (AF) is the most common cardiac arrhythmia with a significant health and economic burden. AF may cause disabling symptoms, often accompanied by multiple comorbidities, is the leading risk factor for stroke and therefore increases the likelihood of mortality [31].

Arterial hypertension was the most common comorbidity in our study, followed by diabetes mellitus, cardiac failure, rheumatological diseases, chronic lung and kidney diseases, previous history of stroke, and liver diseases. The results of our study are observed to be comparable to other studies. Elevated blood pressure was found to be the most important contributor for AF in several studies [32, 33]. An observational, multinational, prospective study from Türkiye (ASPECT‐NOAC) reported that arterial hypertension, coronary artery disease, diabetes mellitus, valvular heart disease, dyslipidemia, chronic obstructive pulmonary disease, and cardiomyopathy were the most common comorbidities [18]. In EORP‐AF, patients aged ≥ 75 years more frequently had a history of hypertension, chronic obstructive pulmonary disease, previous hemorrhagic events, or valvular heart diseases [34].

European Society of Cardiology (ESC) guidelines recommend OACs for men with a CHA2DS2‐VASc Score ≥ 2 and women with a Score ≥ 3 [6]. NOACs are recommended in preference to VKAs except for patients with mechanical heart valves and moderate to severe mitral stenosis [6]. According to the results of an international survey including nonvalvular AF patients under OAC for stroke prevention, the involvement of patients in shared decision making to customize the treatment plan and anticoagulation administration of patients with AF was determined a Class I recommendation in 2014 [24]. In 2021, ESC also recommended to consider patient preferences in AF management in the most recent guideline [6]. Although there are several global reports highlighting clinician and patient preferences associated with OAC selection and dosing, the data in Türkiye is lacking [10, 11, 22, 35]. Such insights can be helpful to ensure that current practice in Türkiye is consistent with patient and physician preferences. This is the first study using BWS to assess patient and physician preferences for OAC treatments in AF in Türkiye.

In our study, efficacy (stroke reduction) and safety (major bleeding) were found to be the most important attributes for the OAC choice in AF for all Turkish participants, whereas the sum of success in preventing stroke and lower risk for major bleeding were 77% and 97.3% of overall importance for patients and physicians, respectively. In line with this, further analysis assuming these two factors being equal among alternatives revealed that the other two safety parameters including the availability of a reversal agent and the need for monitoring as the most important concerns for both groups, whilst from physicians' perspective these attributes gained a higher share of preference compared to patients. In a recent study from the United States, patients and clinicians were asked to assess and rate the importance of 25 factors in anticoagulation decision making. Patients and clinicians ranked stroke prevention and avoiding severe bleeding as very important for the decision of anticoagulation treatment. Patients were more likely to mark the flexibility to decrease anticoagulation dose if needed as a significant criterion (70.5% vs. 43.6%; *p* < 0.001) [25]. A national survey from Canada involved randomly selected patients and physicians including general practitioners, family medicine specialists, cardiologists, and internal medicine specialists to highlight the preferences of patients and physicians regarding OAC therapy. While patients ranked interactions with food and drugs and the availability of a reversal agent as the most important factor for decision, physicians considered the risk for major bleeding at first instance [22]. Similar to our data, another survey including both physicians and patients reported that stroke and bleeding risk were the key elements of treatment selection for both patients and physicians. Dosing frequency was not a priority for both response groups. Physicians also put a notable impact on oral anticoagulants that do not need regular blood checks or dietary limitations [23]. Thus it can be argued that when efficacy/safety and other attributes are considered together as in our study, efficacy/safety takes precedence over other attributes, for both physician and patient preferences.

While safety and efficacy are the main concerns, there are several studies investigating other attributes. A study involving 758 patients from France, Germany, and the United Kingdom revealed that 42.8% of patients primarily prefer a once‐daily anticoagulation therapy. Shorter distance to treating physicians (25.0%), a small‐sized tablet (21.5%), and intake independent of food (10.6%) were the following concerns [36]. The multinational European Patient Survey in Atrial Fibrillation (EUPS‐AF) reported that 80.7% of patients expressed a preference for taking anticoagulation medication once daily, whereas 28% of respondents favored reduced anticoagulation testing as it is time‐saving [35]. A computer‐assisted telephone interview study showed that patients gradually preferred the factors; once daily intake, no need for bridging necessary, close distance to physician, less interactions with food/nutrition, and no/less need for anticoagulation testing for dose adjustment [10]. A cross‐sectional survey of 937 adults with AF receiving OAC for stroke prevention across the United States, Canada, Germany, France, and Japan illustrated that success in stroke prevention was rated as the most significant factor regardless of country of residence, whether they had experienced a recent stroke, and age, sex, or educational level [24]. In the conjoint analysis, stroke prevention was the most rated feature, followed by lower major bleeding risk, less frequent dosing, and application with/without food. Preferences did not change with the extent of stroke knowledge, awareness of AF severity, worry for stroke, or medication burden [37]. Success in stroke prevention and lower risk for bleeding were followed by need for monitoring and availability of antidotes in a web‐based patient survey from Canada [26]. A multicentric discrete choice experiment study compared AF patient attributes for OACs among five different countries. Frequency of intake was the most important concern for German patients while Swiss and Swedish patients determined food/alcohol interaction as a strong criterion. Spanish patients mostly ranked the requirement for monitoring and the need for bridging is the most common influencer among Taiwanese patients in terms of OAC preference [38]. Major bleeding risk followed by minor bleeding risk were the two main concerns for both patients and caregivers in a cross‐sectional study from Spain for the oral anticoagulation decision in AF. Daily dosing frequency was the third‐ranked point by the patients [39]. These results also show that attributes such as medication frequency, testing, or food/drug interactions determine preference only when efficacy and safety are not considered or assumed equal.

In conclusion, “success in stroke prevention” and “major bleeding risk” were found to be the most important attributes of OAC choice in AF in Türkiye. These attributes accounted for 77% and 97.3% of overall importance for patients and physicians, respectively. Certain other treatment considerations, especially reversal agent availability and monitoring, appear to be more important to patients than to physicians. Therefore, to contribute to an improvement of the treatment individualization and to facilitate shared decision making, efficacy, and safety should remain to be main drivers for treatment choice. This is the first study conducted in Türkiye to inform decision‐making by addressing the data gap regarding patient and physician preferences for OAC treatments in AF. In this regard, developing local guidelines or treatment algorithms, taking into account the preferences of both patients and physicians regarding these attributes may increase treatment success, compliance, and adherence.

## 5 Limitations of the Study

The current study has several limitations. First, despite the random selection of study centers, there might be a substantial selection bias arising from physician/patient inclusion. Secondly, there might be a lack of investigated attributes in our surveys. Although BWS quantifies patient priorities in a transparent and accessible manner, there are also some constraints caused by the methodology. Attributes are qualitatively described which may be subjective for respondent interpretation. They may be interpreted differently than intended and there may be variabilities across respondents. To minimize these, questionnaires in our study have been derived from highly ranked attributes in previous studies. Also in the additional analysis, where the top two factors are to be assumed equal and the remaining factors are thus evaluated, it could be argued that the ranking would have changed if the top two factors were never shown in the first place. Even though this argument has some merit in a fictional setting, there is no realistic implication since it is impossible for both practitioners and patients to neglect/exclude these two factors from the decision process.

## Ethics Statement

Ethics committee approval was obtained from Dokuz Eylül University, İzmir, Türkiye. The survey was conducted in accordance with the principles of the Declaration of Helsinki.

## Consent

Written informed consent was obtained from all participants.

## Conflicts of Interest

Kadriye Kılıçkesmez received an honorarium from Pfizer in connection with the development of this manuscript. Dursun Aras received an honorarium from Pfizer in connection with the development of this manuscript. Muzaffer Değertekin received an honorarium from Pfizer in connection with the development of this manuscript. Necla Özer received an honorarium from Pfizer in connection with the development of this manuscript. Oktay Ergene received an honorarium from Pfizer in connection with the development of this manuscript. H.B., H.K., E.D.A., K.U., and O.B. are the employees of Pfizer. Medical writing and editorial support were provided by Ferda Kızıltaş at Remedium Consulting Group and were funded by Pfizer.

## Data Availability

The data that support the findings of this study are available from the corresponding author upon reasonable request. Core data of this study is available via corresponding author upon request.

## Appendix 1

**Variables of interest and content from the approved study protocol by the ethical committee**

Clinicians and patients will be asked to determine the importance of factors regarding the use of anticoagulation in AF by ranking them. Patients and physicians will be ranking the same factors. Although the language may differ considering the difference between professional and lay audiences. Clinicians will also be asked whether their answers change based on five different patient profiles as listed (such as secondary stroke prevention or old age). Patients will also be asked about their perception on their well‐being and medication adherence behaviors. The survey questions for both patients and physicians that will be used in this study can be found in Annex 1.

**Patient Survey**

Variables

- Age
- Gender
- Education
- Socioeconomic status
- Time since AF diagnosis
- Time since anticoagulation initiation
- Number of people in the household
- Prespecified coexisting diseases
  Rank of importance of following factors regarding the perception of the use of anticoagulation
- Success in preventing stroke
- Risk of major bleeding (bleedings that would require admission to emergency care)
- Risk of minor bleeding (bleedings that would not require admission to emergency care, such as gingival bleeding, bruising)
- Daily use frequency
- Availability of treatments that can reverse anticoagulation in case it is necessary (such as bleeding, overdose, etc.)
- Requirement of blood tests in regular intervals
- Restrictions due to food and drug interactions
- How long the drug has been available
- Ease of swallowing
- Whether a drug is required to be taken with food or not
  Patients will also be asked about their perspectives regarding acceptance and control of their well‐being and medication adherence behaviors.
  **Physician Survey**
  Variables
- Experience (years)
- Academic title
- Gender
- Type of institution (secondary care, tertiary care, university, private, etc.)
- Institutions with or without medical education feature
- City
  Rank of the importance of the following factors regarding the perception of use of anticoagulation
- Success in preventing stroke
- Risk of major bleeding (bleedings that would require admission to emergency care)
- Risk of minor bleeding (bleedings that would not require admission to emergency care, such as gingival bleeding, bruising)
- Daily use frequency
- Availability of treatments that can reverse anticoagulation in case it is necessary (such as bleeding, overdose, etc.)
- Requirement of blood tests in regular intervals
- Restrictions due to food and drug interactions
- How long the drug has been available
- Ease of swallowing
- Whether a drug is required to be taken with food or not

Physicians will also be asked whether the ranking changes according to the patient subgroups below, and rankings for the subgroups will be collected in case it is different from the first ranking:

- Secondary stroke prevention
- Elderly patients (patients older than 80 years of age)
- ACS and PCI patients with AF
- Male patients with CHADVASC = 1 or female patients with CHADVASC = 2
- Patients with renal function impairment (GFR < 60)
- Patients with minor bleeding history
