# Correction to “Physician and Patient Preferences for Oral Anticoagulation Therapy Decision Making in Atrial Fibrillation: Results From a National Best–Worst Scaling Survey in Türkiye”

**DOI:** 10.1002/clc.70193

**Published:** 2025-08-21

**Authors:** Pref‐Af Study Group, K. Kılıckesmez, D. Aras, M. Degertekin, N. Ozer, B. Hacibedel, K. Helvacioglu, U. Koc, B. Ozdengulsun, E. Dundar Ahi, Pref‐Af Study Group, O. Ergene

K. Kılıckesmez, D. Aras, M. Degertekin, et al., “Physician and Patient Preferences for Oral Anticoagulation Therapy Decision Making in Atrial Fibrillation: Results From a National Best–Worst Scaling Survey in Türkiye,” *Clinical Cardiology* 47 (2024): e70038. https://doi.org/10.1002/clc.70038

In the published version of this article, the **Pref‐Af Study Group** was missing in the author by‐line. The correct author list should be:

K. Kılıckesmez, D. Aras, M. Degertekin, N. Ozer, B. Hacibedel, K. Helvacioglu, U. Koc, B. Ozdengulsun, E. Dundar Ahi, Pref‐Af Study Group, and O. Ergene

Additionally, the **Acknowledgments** section has been updated as follows:

Medical writing and editorial support was provided by Ferda Kiziltas at remedium Consulting Group. The Pref‐Af study group contributed to this study during the data collection and the names of the contributors as follows: Betul Balaban Kocas, Firdevs Aysenur Ekizler, Ayse Colak, Ahmet Anil Baskurt, Erdal Durmus, and Ugur Nadir Karakulak.

We apologize for this error.

